# Does gonorrhoea screening intensity play a role in the early selection of antimicrobial resistance in men who have sex with men (MSM)? A comparative study of Belgium and the United Kingdom

**DOI:** 10.12688/f1000research.14869.2

**Published:** 2018-08-09

**Authors:** Chris R. Kenyon, Irith De Baetselier, Tania Crucitti

**Affiliations:** 1 HIV/STI Unit, Institute of Tropical Medicine, Antwerp, Belgium; 2 Division of Infectious Diseases and HIV Medicine, University of Cape Town, Cape Town, South Africa; 3 HIV/STI Reference Laboratory, Institute of Tropical Medicine, Antwerp, Belgium

**Keywords:** Neisseria gonorrhoeae, antimicrobial resistance, screening

## Abstract

**Background:** It is unclear why antimicrobial resistance in *Neisseria* *gonorrhoeae* in the United Kingdom (UK) and the United States has tended to first appear in men who have sex with men (MSM). We hypothesize that increased exposure to antimicrobials from intensive STI screening programmes plays a role.

**Methods: **We assess if there is a difference in the distribution of azithromycin, cefixime and ceftriaxone minimum inhibitory concentrations (MICs) between MSM and women in the United Kingdom (UK) where 70% of MSM report STI screening in the past year vs. Belgium where 9% report STI screening in the past year. Our hypothesis is that MICs of the MSM should be higher than those of the women in the UK but not Belgium. Data for the MICs were taken from the Gonococcal Resistance to Antimicrobials Surveillance Programme (GRASP) in the UK in 2010/2011 and 2014 and a similar national surveillance programme in Belgium in 2013/2014 (the first most complete available data). We used the Mann–Whitney test to compare the MIC distributions between MSM and women within each country

**Results:** In the UK the MICs for all three antimicrobials were significantly higher in MSM than women at both time points (P all <0.0005). In Belgium only the MIC distribution for azithromycin was higher in MSM (P<0.0005).

**Conclusion:** The findings for cefixime and ceftriaxone, but not azithromycin are compatible with our hypothesis that screening-intensity could contribute to the emergence of AMR. Numerous other interpretations of our results are discussed.

## Introduction

**Figure 1.**
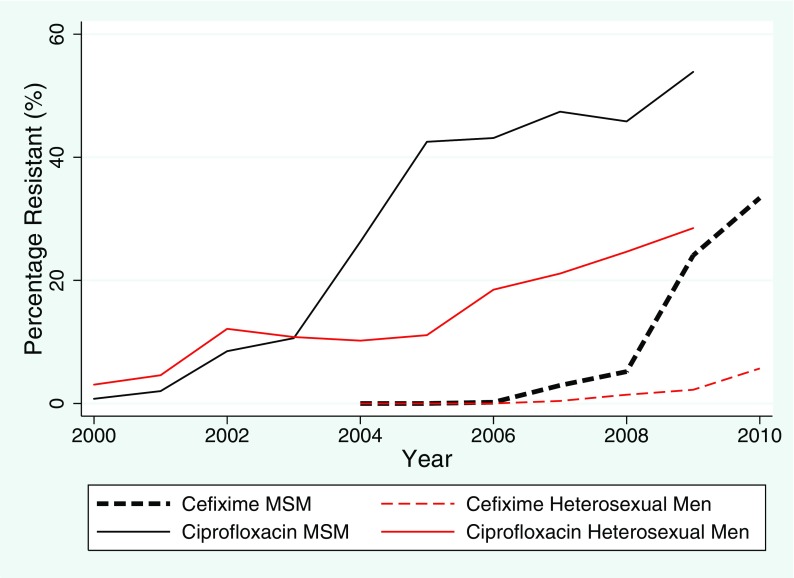
Percent of NG isolates in the United Kingdom showing decreased susceptibility to cefixime and ciprofloxacin in men by sexual orientation 2000–2010 (Based on data from 2, 5).

A striking feature of the patterning of antimicrobial resistance (AMR) is how it has repeatedly emerged in core-groups, either sex workers or men who have sex with men (MSM) with high rates of partner change ^1^. In the last two decades AMR in the United Kingdom (UK) and the United States (USA) has tended to first appear in MSM ^2– 5^. In the UK for example, the prevalence of cefixime resistance (following the switch to cefixime therapy for *Neisseria gonorrhoeae* (NG) in 2005) increased from 0% in 2005 to 33.1% in 2010 in MSM, whilst remaining under 7% in heterosexual men and women ( Figure 1) ^2^. In the USA, UK and the Netherlands, the prevalence of AMR to at least one of ciprofloxacin/cefixime/cefotaxime/azithromycin has been noted to be higher in MSM ^3, 4, 6^. This association has not, however, been found in other countries. An analysis of gonococcal AMR in the 24 countries participating in European Gonococcal Antimicrobial Surveillance Programme (Euro-GASP) in 2015, for example, found that cefixime and ciprofloxacin resistance rates were not higher in MSM compared to heterosexual men ^7^. Azithromycin (AZM) resistance prevalence was however higher in men (both MSM and heterosexuals) than women.

We hypothesize that these differences in the emergence of AMR may be in part explained by differences in the intensity of NG/CT ( *Chlamydia trachomatis*) screening for MSM. The percent of MSM who report being screened for NG/CT varies considerably between countries. In the 38 countries in the European MSM Internet Survey, for example the proportion of MSM who reported anal screening for sexually transmitted infections (STIs) ranged from 4.4% in Serbia to 70.6% in Malta (median 16.0, IQR 13.5-28.4) ^8^. A higher screening intensity would be expected to translate into greater antimicrobial exposure. A study that modelled the sexual network of a population of Belgian MSM, for example, found that increasing screening intensity from 3.5% to 50% of MSM annually would reduce NG prevalence marginally but at the expense of a 12-fold increase in antimicrobial exposure ^8, 9^.

In this preliminary study to test the hypothesis that screening intensity played a role in the selection of AMR in NG we contrast the difference in azithromycin, cefixime and ceftriaxone minimum inhibitory concentration (MIC) distributions between MSM and women in the UK (an intensive-screening country; 70% of MSM report annual STI screening ^8^) with those in Belgium (a low-screening country; 9% of MSM report annual STI screening ^8^) in the years 2010 to 2015. The overall consumption of these antimicrobials in these two countries was not too dissimilar. Between 2010 and 2015, Belgians consumed slightly more cephalosporins but fewer macrolides than the inhabitants of the UK (cephalosporins: 966 vs. 905 standard units per 1000/population/year; macrolides 1960 vs. 3063 standard units per 1000/population/year, respectively ^10^).

### National treatment guidelines for NG in the UK and Belgium

In Belgium, guidelines changed from ciprofloxacin to ceftriaxone 125mg IM or spectinomycin 2g IM in 2008 ^11, 12^. In 2012 azithromycin was added for treatment of NG and ceftriaxone dosage was increased: ceftriaxone 500mg IM plus azithromycin 2g PO ^13, 14^. In the UK, cefixime 400mg PO took over from ciprofloxacin in 2005 as preferred therapy ^3^. In 2011, this was switched to ceftriaxone 500mg IM plus azithromycin 1g PO ^3, 15^. Thus between 2008 and 2012 therapy in Belgium/the UK was mostly ceftriaxone/cefixime whereas from 2012 dual therapy was recommended in both countries.

## Methods

Because the sampling and susceptibility testing methodologies vary slightly between Belgium and the UK, we do not directly compare the MICs between the two countries. Rather we assess if there is a difference in the distribution of MICs between MSM and women in each country. The rationale we use is as follows. If intensive screening in MSM plays a role in generating AMR in MSM then in the intensive-screening country we would expect to find a shift in distribution towards higher MICs in MSM compared to women for the antimicrobials used as treatment in the screening programme. In the low-screening country there should be no difference in distribution between MSM and women. We compare MSM with women rather than heterosexual men to avoid the problem of misclassification of men who occasionally have sex with men but regard themselves as heterosexual ^16^.

**AMR surveillance in Belgium:** All laboratories in Belgium are requested to send NG isolates to the National Reference Centre for STIs (NRC-STI) at the Institute of Tropical Medicine. The agar dilution method was used to determine MICs according to the CLSI guidelines ^17^.

**AMR surveillance in the United Kingdom:** The Gonococcal Resistance to Antimicrobials Surveillance Programme (GRASP) is a sentinel surveillance programme for AMR in NG in the UK. It incorporates a network of genitourinary medicine (GUM) clinics chosen to give regional representation across England and Wales. Isolates from approximately 10% of patients with gonorrhoea, collected over a 3-month period (July–September) each year, undergo susceptibility testing via MIC determination using the agar dilution method at the Public Health England’s sexually transmitted bacteria reference unit (PHE) ^18^. Demographic and behavioural data are gathered retrospectively and then linked to laboratory data ^3^.

### Data sets

The data for Belgium was taken directly from NRC-STI. The details regarding sexual orientation started to be reported in sufficient numbers from 2013 onwards. Because the absolute number of isolates from Belgium are low we present analyses from the combined data from 2013 and 2014.

The data for the UK was extracted from the GRASP annual reports ^2, 5, 18^. This included digitalization of the percent distribution of MIC by sexual orientation/gender graphs using GetData Graph Digitizer 2.26. We analyze the data of 2010 for ceftriaxone and of 2011 for azithromycin and cefixime, as well as the data of 2014 for the three antimicrobials.

For the UK data ethics approval for GRASP was obtained from local regional research committees and from the northwest multicentre research ethics committee ^3^. We used extracted data from publically available reports and thus no additional ethical approval was necessary. In Belgium no additional ethical approval was necessary because only fully anonymized routine surveillance data were used.

### Statistical analyses

We used the Mann–Whitney test to assess if there was a difference in the MIC distributions between MSM and women within each country. Stata 13 was used for all analyses.

## Results

### Belgium

The STI reference laboratory received 1224 NG isolates from 78 laboratories in 2013/2014. Of these, 1150 were successfully cultured and tested. 941 (81.8%) were men, 190 (16.5%) women and 19 unknown gender. 183 (19.5%) of the men reported being heterosexual, 201 (21.4%) MSM and data was missing in 557 (59.2%) men.

**Figure 2.**
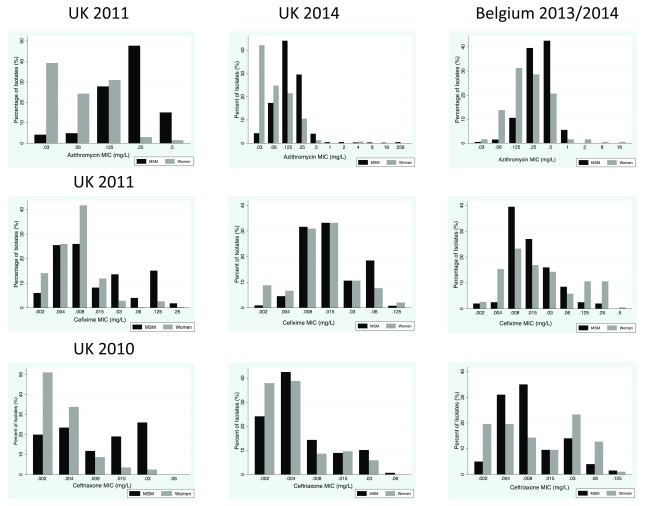
The percent distribution MICs of *Neisseria gonorrhoeae* isolates by gender/sexual orientation in Belgium and the United Kingdom 2010 to 2014.

The distribution of the azithromycin MICs was significantly higher in MSM compared to women (Median MIC 0.25, [IQR 0.25-0.50] vs. 0.25 [0.125-0.25]; P<0.0005) but there were no differences in the MIC distributions for cefixime or ceftriaxone ( Table 1; Figure 2). The MIC distribution for azithromycin was slightly right-shifted in MSM compared to women ( Figure 2). The distribution of the MICs for cefixime in women appeared bimodal, as was the MIC distribution for ceftriaxone in women and to a lesser extent in men.

**Table 1.** MIC distributions for MSM and women in Belgium and the United Kingdom based on data from national reporting systems.

	UK (2010/2011)		UK (2014)		Belgium (2013/2014)	
	MSM (2010: n=600, 2011: n=665)	Women (2010: n=399, 2011: n=387)	MSM (n=1073)	Women (n=192)	MSM (n=200)	Women (n=189)
**Azithromycin** [Median (IQR)]	0.25 (0.125-0.25)	0.06 (0.03-0.125) ***	0.125 (0.125-0.25)	0.06 (0.03-0.125) ***	0.25 (0.25-0.50)	0.25 (0.125-0.25) ***
**Cefixime** [Median (IQR)]	0.008 (0.004-0.03)	0.008 (0.004-0.008) ***	0.015 (0.008-0.03)	0.015 (0.008-0.015) ***	0.015 (0.008-0.03)	0.015 (0.008-0.06)
**Ceftriaxone** [Median (IQR)]	0.008 (0.004-0.03)	0.002 (0.002-0.004) ***	0.004 (0.004-0.008)	0.004 (0.002-0.004) ***	0.008 (0.004-0.015)	0.008 (0.004-0.03)

*** *P*<0.0005 (P-values are from Man-Whitney tests comparing MICs distributions between MSM and women in each country); IQR – Interquartile range

### United Kingdom

The number of isolates provided by the GRASP surveys was as follows: 2010: MSM 600, women 306; 2011: MSM 665, women 312; 2014: MSM 1073, women 192. For further details please refer to the individual annual reports ^2, 5, 18^.

***2010–2011:*** The MIC distributions for all three antimicrobials were statistically significantly higher in MSM than women (Azithromycin: 0.25, [IQR 0.125-0.50] vs. 0.06 [0.03-0.125], cefixime: 0.008, [IQR 0.004-0.03] vs. 0.002 [0.002-0.004] ceftriaxone: 0.008, [IQR 0.004-0.03] vs. 0.008 [0.004-0.008]; All P<0.0005). For all three antimicrobials the distribution was right-shifted in MSM compared to women ( Figure 2). The distributions of the MICs for cefixime and ceftriaxone in MSM appeared bimodal.

***2014:*** The MIC distributions for all three antimicrobials were statistically significantly higher in MSM compared to women and were shifted to the right but less so than in 2010 or 2011 ( Figure 2, Table 1).

The distributions of the MICs for cefixime in MSM appeared bimodal, but with a shift to the left of the second mode compared to 2011. The bimodal appearance of the MIC distribution for ceftriaxone in 2014 is less pronounced compared to 2010.

Minimum inhibitory concentrations distributions for Neisseria gonorrhoeae isolates analyzed Click here for additional data file. Copyright: © 2018 Kenyon CR et al. 2018 Data associated with the article are available under the terms of the Creative Commons Zero "No rights reserved" data waiver (CC0 1.0 Public domain dedication).

## Discussion

A better understanding of the factors underpinning the genesis of AMR in NG could assist with efforts to prevent the further development of AMR. In this study we find that the MIC distribution for azithromycin, ceftriaxone and cefixime (particularly in 2010) is right shifted in MSM compared to women in the UK. In Belgium only the distribution of azithromycin is right-shifted in this way. In addition, we find that the magnitude of this right-shift decreased in the UK between 2010/2011 and 2014. As a result, the proportion of MSM in the UK with higher ceftriaxone MICs and cefixime MICs has declined between 2010 and 2014. These findings are commensurate with UK and European surveillance data showing a decline in the proportion of third generation cephalosporin AMR ^2, 7, 15^. A plausible reason for this decline has been the introduction of high dose ceftriaxone which has more favourable pharmacokinetic parameters than cefixime ^2, 3, 19^. Dual therapy with azithromycin may also have played a role ^7, 20^.

What explains the right-shifting of cefixime and ceftriaxone in MSM versus women in the UK but not Belgium? An important difference in the pharmacoecology experienced by NG in the two countries was the use of cefixime in the UK (until 2011) compared to ceftriaxone monotherapy in Belgium (until 2012). Ceftriaxone's longer half-life than cefixime may have played a role in preventing MIC drift in Belgium ^19^. For a number of bug-drug combinations cefixime has been found to be more prone to AMR than other third generation cephalosporins. One of the most convincing studies of this was an *in vitro* differential selection study by Negri *et al*., who found that cefixime was the best selector of penicillin resistance in *Streptococcus pneumoniae* (compared to amoxicillin, cefuroxime and cefotaxime ^21^. The mechanism underpinning this effect has not been clearly elucidated but a number of authors have speculated that it may be related at least in part to cefixime's shorter half life. Both women and men were treated with cefixime in the UK and this would thus not explain why the right-shifting occurred predominantly/only in MSM. The higher NG screening (and therefore antibiotic exposure rates) in MSM in the UK compared to Belgium is one of many possible explanations. This explanation stems from the insight that the intensity of exposure to antimicrobials plays a crucial role in the genesis of AMR ^22– 26^. A range of studies have found close correlations at ecological levels between the intensity of exposure to a particular antimicrobial and AMR to that antimicrobial ^22– 24, 27, 28^. These findings have led us and others to propose the pharmacoecological theory of AMR (connectivity AMR theory) which posits that it is the combination of dense sexual networks plus excess antimicrobial consumption (such as from intense screening) that plays an important role in AMR genesis in NG ^29^. The dense sex network generates the high prevalence of NG and the antimicrobial exposure then initially lowers prevalence but in the process generates a fitness advantage for resistant NG.

Arguing against the screening-intensity explanation is the fact that the right shifting of AZM occurred in MSM in both countries. This finding suggests either that some other factor is responsible for the right shifting in MSM (such as total macrolide use for all indications) or that the MSM sexual pharmacoecology is more susceptible to the development of AMR for azithromycin than other antibiotics ^30^. The higher proportion of time NG spends in the rectum in MSM compared to heterosexual sexual networks, for example, could lead to an enhanced selection pressure for/availability of *mtrR*-related and *erm* mutations ^30^. Macrolides have also been shown to have a particularly long adverse effect on the resistome, with changes noted for up to 4 years post therapy ^31, 32^. These considerations may mean that relatively low azithromycin exposure may be sufficient to generate a right shift in MIC. Gonorrhoea screening guidelines for MSM attending specialist sexual health services in the UK were updated in 2010, and this was followed by an increase in NG tests since then. Since cephalosporin resistance rates in the UK declined post 2010, this evidence is not supportive of the screening-intensity hypothesis.

We also observed changes in the bimodal distribution of cetriaxone and cefixime in 2014 versus 2010–2011 in the UK. The shift to the left of the second mode and almost disappearance of the bimodal distribution is reassuring as it may indicate that the previous emergence of a less susceptible population is temporarily under control and regaining susceptibility towards cefixime and ceftriaxone.

There are a number of alternative explanations for why AMR may arise sooner in MSM than women. MSM may be more likely to travel abroad and acquire more resistant NG in this way ^4, 33^. At least one study has however found that heterosexuals with gonorrhoea are more likely to report sex abroad than MSM ^33^. MSM are more likely to be HIV-infected and may as a result use more antimicrobials ^4^. Some studies have found that even after stratifying for HIV-infection status, MSM still report consuming more antimicrobials ^4^. Both treatment of symptomatic and asymptomatic STIs may play a role here. Finally, the fact that NG spends proportionately more time in the oropharynx and rectum in MSM (compared to heterosexuals) may offer it more opportunities for acquisition of resistance genes and mutations ^4, 30^. It is however unlikely that these explanations can explain the differences between NG AMR in MSM vs. women in the UK compared to Belgium.

The numerous weaknesses of our study design preclude firm conclusions. These limitations include the fact that we only include two countries, and we have limited data on the full range of potential explanatory variables (such as general antimicrobial consumption, NG/CT screening rates in women). There were also important methodological differences in how the surveillance was conducted in the two countries (such as sampling methodology, sensitivity testing). Whereas the GRASP sentinel methodology has been shown to yield fairly representative samples for the UK ^34^, an equivalent study has not been conducted in Belgium.

Although we cannot, on the basis of this study, conclude that the intensity of NG screening plays a role in the genesis of AMR in NG we also cannot reject this hypothesis. Further studies that could test this hypothesis include: 1) assessing the correlation between NG screening intensity in MSM and the prevalence of AMR in MSM in a greater number of countries; 2) community level randomized controlled trials assessing the impact of NG/CT screening on AMR and NG prevalence and 3) more detailed longitudinal assessments of the effects of repeated antibiotic exposure on the resistome and microbiome of MSM cohorts with higher risk behaviour ^35^.

## Data availability

The data referenced by this article are under copyright with the following copyright statement: Copyright: © 2018 Kenyon CR et al.

Data associated with the article are available under the terms of the Creative Commons Zero "No rights reserved" data waiver (CC0 1.0 Public domain dedication). 

Dataset 1: Minimum inhibitory concentrations distributions for *Neisseria gonorrhoeae* isolates analyzed 10.5256/f1000research.14869.d203173 ^36^
