# Supplementary material for: Does gonorrhoea screening intensity play a role in the early selection of antimicrobial resistance in men who have sex with men (MSM)? A comparative study of Belgium and the United Kingdom
Source: F1000Res. 2018 Aug 9;7:569. Originally published 2018 May 10. [Version 2] doi: 10.12688/f1000research.14869.2 (PMC6192441; doi:10.12688/f1000research.14869.2)
Supplement: Peer review history

# Peer review: Referee response for version 2

**DOI:** 10.5256/f1000research.17211.r36965

**Published:** 2018-10-15

**Authors:** Hamish Mohammed, Michelle J Cole

**Affiliations:** 1 HIV & STI Department, National Infection Service, Public Health England, London, UK; 2 Antimicrobial Resistanceand Healthcare Associated Infections Reference Unit (AMRHAI), National Infection Service, Public Health England, London, UK

We thank the authors for their revised version. While the proposed hypothesis is of merit, we believe the analyses are not appropriate to assess it due to the inherent biases of an ecologic analysis.

 We would suggest reframing the analysis to focus on data from Belgium, as the authors have access to the raw data and could possibly compare between MSM and heterosexuals (assuming differential intensity of GC screening) with these data.

 We also believe some of our previous comments were not adequately addressed:

- Comment 2 (comparing MSM and heterosexual men)
  - We disagree; there are marked differences between heterosexual men and women in the GRASP reports in respect to azithromycin resistance. In recent years, azithromycin resistance has been very similar in MSM and heterosexual men, which does not support your hypothesis.
- Comment 6 (UK screening guideline update in 2010 leading to an upturn in screening in MSM with a decline in cephalosporin resistance)
  - As mentioned previously*, the upturn in GC screening coincided with a rapid decline in cephalosporin resistance – this also does not support your hypothesis.

* *In the UK, gonorrhoea screening guidelines for MSM attending specialist sexual health services were updated in 2010, and this has led to an increase in gonorrhoea tests since then. This testing trend should be considered when interpreting these findings, as the level of testing prior to 2010 would be much lower than those of more recent years. Furthermore, the upturn in screening coincided with a rapid decline in cephalosporin resistance. *

- EMIS is an excellent source of survey data from a community sample but, as you acknowledge, there may be some sampling bias between countries. Are there STI surveillance data from Belgium to indicate how many MSM are tested for GC and, if not, can we confidently conclude that MSM are more likely to be screened for GC in the UK vs. Belgium?
- Also, in reference to your response: we would still argue that 6% and 8.3% from the Belgium national data is still a lot higher than the 1.7% and 0.4% cefixime resistance data from GRASP.
- In the UK, AMR is often imported from Asia and has spread and developed in MSM, but has not necessarily first emerged in MSM.
- The analyses of GRASP data are based on numbers extrapolated from figures using an online tool; has this tool been validated?
- Another limitation is the lack of data from Belgium in 2009/10 as we feel a similar shift in MICs in men would have been observed due to the international spread of ST1407.
- While sexual orientation data isn’t available, could you examine gender differences instead?
- The GRASP ethics statement is still incorrect.

We have read this submission. We believe that we have an appropriate level of expertise to state that we do not consider it to be of an acceptable scientific standard, for reasons outlined above.

# Peer review: Referee response for version 2

**DOI:** 10.5256/f1000research.17211.r39025

**Published:** 2018-10-15

**Authors:** Xiang-Sheng Chen

**Affiliations:** 1 National Center for STD Control, Chinese Center for Disease Control and Prevention, Nanjing, China

This is an interesting and well-organized article to compare GC AMR (MIC) distributions among men who have sex with men (MSM) and women between the UK and Belgium and conclude the difference in the distribution patterns is due to intensity of screening for sexually transmitted infections. On the current version of the manuscript, I made a few additional comments as follows for authors’ considerations.

  

1. The title refers to “gonorrhoea screening” but I could not find any information/data on gonorrhoea screening but the screening for STI in the manuscript. It may be better to change to “screening intensity for sexually transmitted infections” or “Differences in population-specific gonococcal antimicrobial resistance distributions between the United Kingdom and Belgium” and then use the screening intensity to explain the differences in text.
2. From Belgium part in the Figure 2, we can find a higher MIC distributions for cefixime and ceftriaxone among women than MSM - in contrast to what observed in the UK. Does it mean an increased intensity of screening for STI among women in Belgium? This should be explained in the Discussion.
3. Although screening intensity can be used as proxy of intensity of antimicrobial use/exposure, the further studies indicated in the last paragraph of Discussion should include the assessment of corrections between intensity of the specific antibiotic use and the prevalence of antimicrobial resistance of NG.

I have read this submission. I believe that I have an appropriate level of expertise to confirm that it is of an acceptable scientific standard.

# Peer review: Referee response for version 2

**DOI:** 10.5256/f1000research.17211.r36964

**Published:** 2018-08-10

**Authors:** Henry J C de Vries

**Affiliations:** 1 University of Amsterdam, department of dermatology, Amsterdam Institute for Infection and Immunity (AI&II), Meibergdreef 9, Amsterdam, The Netherlands; 2 Public Health Service (GGD) , Amsterdam, The Netherlands

The manuscript has sufficiently improved. A last comment to increase clarity in figure 2, please add a subheading indicating the time and year of the data depicted above each graph, instead above the top row and left column only.

I have read this submission. I believe that I have an appropriate level of expertise to confirm that it is of an acceptable scientific standard.

# Peer review: Referee response for version 1

**DOI:** 10.5256/f1000research.16184.r35298

**Published:** 2018-07-20

**Authors:** Hamish Mohammed, Jane Hallinan, Michelle J Cole

**Affiliations:** 1 Antimicrobial Resistanceand Healthcare Associated Infections Reference Unit (AMRHAI), National Infection Service, Public Health England, London, UK; 2 HIV & STI Department, National Infection Service, Public Health England, London, UK; 3 Public Health England, London, UK

This is an interesting article that tests the hypothesis that the emergence of gonococcal AMR in MSM is due to increased intensity of screening for gonorrhoea (GC).

 The authors’ findings are based primarily on an ecological analysis and, if these data are available in Belgium, we believe an analysis considering the GC testing history of each person whose MIC data are included would yield useful results.

 We would be keen to discuss the proposed hypothesis and interpretation of GRASP data with you, and have included comments for the authors’ consideration:

1. The evidence for different levels of screening intensity in MSM is available from EMIS; is there a comparable source of these data for heterosexual women? Also, did the recruitment strategy to participate in EMIS vary between the UK and Belgium? If so, this would meant that response to the question on the history of STI screening may be less comparable between these two countries.
2.  We believe a comparison of MSM with heterosexual men may be more useful, even with the limitations of underreporting of same-sex contact in heterosexually-identifying men. This is because the numbers of isolates are more comparable, women sometimes have different antimicrobial susceptibility profiles from heterosexual men and the cultures available from women are not representative of the circulating isolates in the community due to the difficulty in culturing from women.   
3. In England, most gonorrhoea and chlamydia are diagnosed in people under the age of 25 years, with over one million chlamydia tests conducted annually though the National Chlamydia Screening Programme (NCSP) for 15 to 24 year olds. NCSP testing coverage is more than twice as high in women (28%) than men (11%) of that age-group (data here). Additionally, dual (CT/GC) NAAT platforms are commonly used for the NCSP and, while the positive predictive value of a gonorrhoea test in a community sample is very low, people with false positive results may be incorrectly prescribed antibiotics to treat gonorrhoea. These two aspects may work against the screening intensity hypothesis.
4.  There was no evidence of an association between azithromycin resistant NG and being diagnosed previously with chlamydia or gonorrhoea (as discussed in this paper, which the authors also cite: Clifton et al ^1^); ths suggests that those who get tested for STIs more frequently do not have higher azithromycin MICs.  
5. The authors only considered GRASP data from 2010, 2011 and 2014, but this analysis could be strengthened by including data from more years, including more recently published data: https://www.gov.uk/government/publications/gonococcal-resistance-to-antimicrobials-surveillance-programme-grasp-report. Alternatively, could the authors please specify these three years were selected, or why data from different countries aren’t compared within the same years? Also, could you please clarify why the MIC distributions for all three antibiotics were not analysed for the three time points? For example, the ceftriaxone data from the UK in 2011 could have been considered. In addition, cefixime resistance was widespread across Europe in 2010 due to the ST1407 clone. Cefixime resistance in Belgium was similar than the UK in 2010 according to the Euro-GASP data ( ^2^) and it would not be surprising if the burden of this resistance was in MSM in Belgium also. This analysis would be strengthened by including Belgium data from 2010/2011. 
6.  Interestingly, Euro-GASP 2015 and 2016 data show much higher cefixime resistance in Belgium than the UK. According to the proposed hypothesis, should the opposite pattern have been observed? It would also be interesting to speculate the level of cefixime resistance in MSM in the UK if asymptomatic, multiple-site screening was not in place.  
7. In the UK, gonorrhoea screening guidelines for MSM attending specialist sexual health services were updated in 2010, and this has led to an increase in gonorrhoea tests since then. This testing trend should be considered when interpreting these findings, as the level of testing prior to 2010 would be much lower than those of more recent years. Furthermore, the upturn in screening coincided with a rapid decline in cephalosporin resistance.
8.  Between 2010 (gonorrhoea screening guideline) and 2012 (change to dual therapy), the increased screening at sexual health services would have detected more cases and these were treated with cefixime which has been shown to be less effective, particularly at the pharynx (Barbee 2013). This would suggest that the increased prevalence of cefixime resistance in MSM may have been due to the usage of an antibiotic which is less effective than ceftriaxone, rather than the result of intensive screening.   
9. Figure 1 shows ciprofloxacin resistance was higher initially in heterosexual men, rather than MSM; therefore, resistance does not always emerge in MSM first.
10.  The labelling of the graphs in figure 2 is unclear – could this be clarified?  
11. In the discussion and in reference to the link between MSM and travel – this may be the case, but we have found no data to support this: Town et al ^3^. In addition one of the references cited mentions that heterosexual men reported more sex abroad than MSM - Matteelli et al ^4^.
12.  As you have used publicly available GRASP data collected for routine surveillance purposes, the ethics statement should be amended accordingly. Similarly, references to the ‘STBRU’ should be to ‘PHE’.  
13. As sexual orientation data was missing for 59% of the Belgium isolates, what are your views on how representative the sample of MSM whose data are considered in this analysis is?
14.  As many of the MIC medians were the same and some of MIC distributions that are declared as significantly different do not appear very different on inspection, particularly for 2014 UK cefixime and ceftriaxone, it would be useful if additional analysis were performed, such as linear regression with the geometric MIC means.

We have read this submission. We believe that we have an appropriate level of expertise to confirm that it is of an acceptable scientific standard, however we have significant reservations, as outlined above.

## References

[1] CliftonSTownKFuregatoMColeMMohammedHWoodhallSCKevin DunbarJFiferHHughesG: Is previous azithromycin treatment associated with azithromycin resistance in Neisseria gonorrhoeae? A cross-sectional study using national surveillance data in England. *Sex Transm Infect*.2018; 10.1136/sextrans-2017-053461 10.1136/sextrans-2017-053461 29511067

[2] ColeMQuayeNChisholmSIsonCvan de LaarMSpiteriG: Gonococcal antimicrobial susceptibility surveillance in Europe 2010. *ECDC*.2012; 10.2900/36167 10.2900/36167

[3] TownkFieldNColeMHarrisSHughesG: What role does importation play in the spread of antimicrobial resistant neisseria gonorrhoeae in the uk? associations between antimicrobial resistant strains and recent sex abroad. *BMJ journals*.2017;93(2) : 10.1136/sextrans-2017-053264.26 10.1136/sextrans-2017-053264.26

[4] MatteelliACarosiG: Sexually transmitted diseases in travelers. *Clin Infect Dis*.2001;32(7) : 10.1086/319607 1063-7 10.1086/319607 11264035

# Response

**Published:** 2018-07-26

**Authors:** Chris Kenyon

**Affiliations:** Institute of Tropical Medicine, Antwerp, Belgium

We would be keen to discuss the proposed hypothesis and interpretation of GRASP data with you, and have included comments for the authors’ consideration:

**Reply:**

**Thank you for your interest and most useful comments. We would be most interested in collaborating on further studies along these lines.**

 

1. The evidence for different levels of screening intensity in MSM is available from EMIS; is there a comparable source of these data for heterosexual women? Also, did the recruitment strategy to participate in EMIS vary between the UK and Belgium? If so, this would meant that response to the question on the history of STI screening may be less comparable between these two countries.

 

**Reply:**

**We looked and unfortunately could not find a comparable source for women. The recruitment strategies for EMIS were similar in the two countries. Because EMIS did not collect nationally representative samples we cannot exclude the possibility of a sampling bias that differed between the UK and Belgium. The large numbers recruited in EMIS in both countries and the fact that the results for the screening and other questions are commensurate with other data sources however argues against such a bias.**

 

1.  We believe a comparison of MSM with heterosexual men may be more useful, even with the limitations of underreporting of same-sex contact in heterosexually-identifying men. This is because the numbers of isolates are more comparable, women sometimes have different antimicrobial susceptibility profiles from heterosexual men and the cultures available from women are not representative of the circulating isolates in the community due to the difficulty in culturing from women.   

 

**Reply:**

**Repeating the analyses with heterosexual men instead of women makes very little difference to the results. As we note above, visual comparisons of the MIC frequency distributions for azithromycin, cefixime and ceftriaxone between heterosexual men and women in the annual GRASP reports reveal little difference. We would be interested to repeat this analysis in using a larger number of countries/subpopulations as outlined in the conclusion.**

 

1. In England, most gonorrhoea and chlamydia are diagnosed in people under the age of 25 years, with over one million chlamydia tests conducted annually though the National Chlamydia Screening Programme (NCSP) for 15 to 24 year olds. NCSP testing coverage is more than twice as high in women (28%) than men (11%) of that age-group (data here). Additionally, dual (CT/GC) NAAT platforms are commonly used for the NCSP and, while the positive predictive value of a gonorrhoea test in a community sample is very low, people with false positive results may be incorrectly prescribed antibiotics to treat gonorrhoea. These two aspects may work against the screening intensity hypothesis.

 

**Reply:**

**This is a useful observation. We have thought at some length about how screening intensity may produce antimicrobial resistance (AMR) in N. gonorrhoeae (Ng). This has lead to the pharmacoecological theory of AMR (connectivity AMR theory) which posits that it is the combination of dense sexual networks plus excess antimicrobial consumption (such as from intense screening) which is responsible. The dense sex network generates the high prevalence of Ng and the antimicrobial exposure then initially lowers prevalence but in the process generates a fitness advantage for resistant Ng** **[4]** **. If this theory is correct then intensive screening in the general heterosexual population in the UK (with its low connectivity network) would not have the same effect on selecting for resistance because the prevalence of Ng is low. Populations of sex workers would also be predicted to be at risk for the genesis of AMR, which has been observed. We have added a section in the discussion to make this clearer. These considerations are best understood by means of the following diagram (Figure 1) which is taken from our recent paper on the topic** **[4]** **:**

URL of Figure 1: https://wwwnc.cdc.gov/eid/article/24/7/17-2104-f2

**Figure 1**.  High network connectivity combined with excess antimicrobial drug exposure from *N. gonorrhoeae* pre-exposure prophylaxis could produce antimicrobial resistance. A dense sexual network translates into a high equilibrium prevalence of *N. gonorrhoeae* (red squares) at time-point 1. Active *N. gonorrhoeae* screening of 50% of this population every 3 months results in 50% lower *N. gonorrhoeae* prevalence at time-point 2 (3 months later) but at the expense of an altered resistome (A _Scr_; black squares represent 3 patients with *N. gonorrhoeae* cleared by screening/treatment. The unchanged underlying network connectivity results in a force that pushes *N. gonorrhoeae* back toward its equilibrium prevalence, placing recently cured patients at high risk for reinfection at a time when their resistomes are enriched with resistance genes. Early reinfecting *N. gonorrhoeae* take up these resistance genes by transformation. In the absence of screening and excess antimicrobial drug use (A _NoScr_) *N. gonorrhoeae* prevalence would not decline but there would be no pressure to select for antimicrobial resistance. Gray squares indicate uninfected persons; lines represent sexual relationships.

 

1.  There was no evidence of an association between azithromycin resistant NG and being diagnosed previously with chlamydia or gonorrhoea (as discussed in this paper, which the authors also cite: Clifton et al 1); ths suggests that those who get tested for STIs more frequently do not have higher azithromycin MICs.  

 

**Reply:**

**We found the paper by Clifton et al. most interesting but are also aware of the paper by Wind et al., which found that receipt of azithromycin in the previous 30 days was associated with an increased MIC** **[5]** **. More important however is the ecological perspective. It is plausible that excess AMR is exerting its effect at the population level. Thus if population A has 30 fold higher macrolide consumption than population B we know from a range of studies that macrolide resistance in *S. pneumonia* and numerous other pathobionts is much more likely to emerge in population A than B. This has been clearly shown in ecological level studies** **[6-8]** **. The association may be harder to establish at an individual level but if one thinks from a pharmacoecologic perspective (and includes considerations of how Ng can acquire AMR from Neisseriaceae and other commensals as it transits through a population) it is easy to see why this is the case** **[4]** **. The deleterious effect of macrolides on the resistome at an individual level have been long established** **[7]** **. These considerations are of considerable importance given the threat of untreatable Ng. We have recently calculated the macrolide and cephalosporin exposure that 3 site** **, 3 monthly screening for gonorrhoea and chlamydia places on PrEP cohort (Unpublished results). We did this via conducting a literature review of the incidence of gonorrhoea and chlamydia in PrEP studies that conducted 3 site, 3 monthly screening. We found that screening results in macrolide consumption rates that considerably exceed those in high macrolide consumption populations where consumption has been strongly associated with macrolide resistance.**

 

1. The authors only considered GRASP data from 2010, 2011 and 2014, but this analysis could be strengthened by including data from more years, including more recently published data: https://www.gov.uk/government/publications/gonococcal-resistance-to-antimicrobials-surveillance-programme-grasp-report. Alternatively, could the authors please specify these three years were selected, or why data from different countries aren’t compared within the same years? Also, could you please clarify why the MIC distributions for all three antibiotics were not analysed for the three time points? For example, the ceftriaxone data from the UK in 2011 could have been considered. In addition, cefixime resistance was widespread across Europe in 2010 due to the ST1407 clone. Cefixime resistance in Belgium was similar than the UK in 2010 according to the Euro-GASP data ( 2) and it would not be surprising if the burden of this resistance was in MSM in Belgium also. This analysis would be strengthened by including Belgian data from 2010/2011. 

 

**Reply:**

**We agree it is frustrating not to have data from Belgium in 2010/11. As we point out in the methods section we could not use this data as we do not have data as to sexual orientation in sufficient number from this period. In the methods we state:**

**The details regarding sexual orientation started to be reported in sufficient numbers from 2013 onwards**

**For the UK data we agree it would be interesting to look at the data from all the available years. We chose the first year at or after 2010 when antimicrobial MIC frequency distributions were reported by gender/sexual orientation. This was 2010 for ceftriaxone and 2011 for azithromycin and cefixime. We then looked at all 3 antimicrobials in 2014 so as to use the same time period for the comparison with Belgium. We considered that this analysis was sufficient for our purposes but acknowledge the reviewers point that there would be a multiplicity of other ways of doing this analysis. Looking at the MIC distributions from other years we consider it likely that this would not substantially change the results. Our analysis involved comparing MIC distributions between MSM and women in the two countries and not comparing MSM or women between the two countries.**

 

1.  Interestingly, Euro-GASP 2015 and 2016 data show much higher cefixime resistance in Belgium than the UK. According to the proposed hypothesis, should the opposite pattern have been observed? It would also be interesting to speculate the level of cefixime resistance in MSM in the UK if asymptomatic, multiple-site screening was not in place.  

 

**Reply:**

**This is an excellent point. The cefixime resistance figures in Belgium 2015/2016 are remarkably high according to the Euro GRASP figures but it must be remembered that these figures are based on a small sample of all national samples for this time period. The full results for 2016 are 597 isolates tested of which 36 (6%°/1(0,1%) had decreased sensitivity to cefixime according to EUCAST/CLSI breakpoints. The results in 2015 were similar: 630 isolates tested with 52 (8,3%)/1(0,1%) classified as decreased sensitivity. These lower rates of resistance are a more accurate representation than the Euro GRASP figures** **[3]** **.**

< >In the UK, gonorrhoea screening guidelines for MSM attending specialist sexual health services were updated in 2010, and this has led to an increase in gonorrhoea tests since then. This testing trend should be considered when interpreting these findings, as the level of testing prior to 2010 would be much lower than those of more recent years. Furthermore, the upturn in screening coincided with a rapid decline in cephalosporin resistance. Between 2010 (gonorrhoea screening guideline) and 2012 (change to dual therapy), the increased screening at sexual health services would have detected more cases and these were treated with cefixime which has been shown to be less effective, particularly at the pharynx (Barbee 2013). This would suggest that the increased prevalence of cefixime resistance in MSM may have been due to the usage of an antibiotic which is less effective than ceftriaxone, rather than the result of intensive screening. Figure 1 shows ciprofloxacin resistance was higher initially in heterosexual men, rather than MSM; therefore, resistance does not always emerge in MSM first. The labelling of the graphs in figure 2 is unclear – could this be clarified?  In the discussion and in reference to the link between MSM and travel – this may be the case, but we have found no data to support this: Town et al 3. In addition one of the references cited mentions that heterosexual men reported more sex abroad than MSM - Matteelli et al 4. As you have used publicly available GRASP data collected for routine surveillance purposes, the ethics statement should be amended accordingly. Similarly, references to the ‘STBRU’ should be to ‘PHE’.  As sexual orientation data was missing for 59% of the Belgium isolates, what are your views on how representative the sample of MSM whose data are considered in this analysis is? As many of the MIC medians were the same and some of MIC distributions that are declared as significantly different do not appear very different on inspection, particularly for 2014 UK cefixime and ceftriaxone, it would be useful if additional analysis were performed, such as linear regression with the geometric MIC means.https://doi.org/10.3201/eid2407.172104.

**Reply:**

**Thanks for this information which is indeed relevant and has been included as a major caveat in the discussion.**

 

1.  Between 2010 (gonorrhoea screening guideline) and 2012 (change to dual therapy), the increased screening at sexual health services would have detected more cases and these were treated with cefixime which has been shown to be less effective, particularly at the pharynx (Barbee 2013). This would suggest that the increased prevalence of cefixime resistance in MSM may have been due to the usage of an antibiotic which is less effective than ceftriaxone, rather than the result of intensive screening. 

 

**Reply:**

**We agree this is a possibility and have expanded the discussion section to reflect his point.**

  

1. Figure 1 shows ciprofloxacin resistance was higher initially in heterosexual men, rather than MSM; therefore, resistance does not always emerge in MSM first.

 

**Reply:**

**This is true as is the repeated emergence of AMR in sex workers and their contacts.**

 

1.  The labelling of the graphs in figure 2 is unclear – could this be clarified?  

 

**Reply:**

**We have asked the publisher to increase the size of the figure to a single page so as to make it easier to read the labeling.**

 

1. In the discussion and in reference to the link between MSM and travel – this may be the case, but we have found no data to support this: Town et al 3. In addition one of the references cited mentions that heterosexual men reported more sex abroad than MSM - Matteelli et al 4.

 

**Reply:**

**Thanks. We have added a sentence pointing this out in the discussion.**

 

1.  As you have used publicly available GRASP data collected for routine surveillance purposes, the ethics statement should be amended accordingly. Similarly, references to the ‘STBRU’ should be to ‘PHE’.  

 

**Reply:**

**Both these changes have been made.**

 

1. As sexual orientation data was missing for 59% of the Belgium isolates, what are your views on how representative the sample of MSM whose data are considered in this analysis is?

 

**Reply:**

**This is a major limitation and as we note in the limitations section means we need to be cautious in any conclusions we draw from this study.**

 

1.  As many of the MIC medians were the same and some of MIC distributions that are declared as significantly different do not appear very different on inspection, particularly for 2014 UK cefixime and ceftriaxone, it would be useful if additional analysis were performed, such as linear regression with the geometric MIC means.

 

**Reply:**

**Numerous other analyses could be done but this is a very simple and limited analysis. We believe that given the major limitations of the study described above and more fully in the paper, it would be inappropriate to conduct further complicated statistical analyses. The data suggest some differences in the relationship between MIC distributions between the 2 countries. The next step should be to find better and more extensive datasets to evaluate further test the hypothesis. We conclude the paper with a description of what we think these analyses could be.**

References:

1.         Chisholm SA, Mouton JW, Lewis DA, Nichols T, Ison CA, Livermore DM. Cephalosporin MIC creep among gonococci: time for a pharmacodynamic rethink? J Antimicrob Chemother. 2010;65(10):2141-8. Epub 2010/08/10. doi: 10.1093/jac/dkq289. PubMed PMID: 20693173.

2.         Negri MC, Morosini MI, Loza E, Baquero F. In vitro selective antibiotic concentrations of beta-lactams for penicillin-resistant Streptococcus pneumoniae populations. Antimicrob Agents Chemother. 1994;38(1):122-5. Epub 1994/01/01. PubMed PMID: 8141563; PubMed Central PMCID: PMCPMC284406.

3.         Cole MJ, Spiteri G, Jacobsson S, Woodford N, Tripodo F, Amato-Gauci AJ, et al. Overall Low Extended-Spectrum Cephalosporin Resistance but high Azithromycin Resistance in Neisseria gonorrhoeae in 24 European Countries, 2015. BMC Infect Dis. 2017;17(1):617. Epub 2017/09/13. doi: 10.1186/s12879-017-2707-z. PubMed PMID: 28893203; PubMed Central PMCID: PMCPMC5594611.

4.         Kenyon C, Schwartz IS. A combination of high sexual network connectivity and excess antimicrobial usage induce the emergence of antimicrobial resistance in Neisseria gonorrhoeae Emerg Infect Dis. 2018;24(7). doi: https://doi.org/10.3201/eid2407.172104.

5.         Wind CM, de Vries E, Schim van der Loeff MF, van Rooijen MS, van Dam AP, Demczuk WH, et al. Decreased azithromycin susceptibility of Neisseria gonorrhoeae isolates in patients recently treated with azithromycin. Clin Infect Dis. 2017. Epub 2017/04/04. doi: 10.1093/clinid/cix249. PubMed PMID: 28369420.

6.         Goossens H, Ferech M, Vander Stichele R, Elseviers M, Group EP. Outpatient antibiotic use in Europe and association with resistance: a cross-national database study. Lancet. 2005;365(9459):579-87. Epub 2005/02/15. doi: 10.1016/S0140-6736(05)17907-0. PubMed PMID: 15708101.

7.         Malhotra-Kumar S, Lammens C, Coenen S, Van Herck K, Goossens H. Effect of azithromycin and clarithromycin therapy on pharyngeal carriage of macrolide-resistant streptococci in healthy volunteers: a randomised, double-blind, placebo-controlled study. The Lancet. 2007;369(9560):482-90.

8.         Garcia-Rey C, Aguilar L, Baquero F, Casal J, Dal-Re R. Importance of local variations in antibiotic consumption and geographical differences of erythromycin and penicillin resistance in Streptococcus pneumoniae. J Clin Microbiol. 2002;40(1):159-64. Epub 2002/01/05. PubMed PMID: 11773111; PubMed Central PMCID: PMCPMC120130.

# Peer review: Referee response for version 1

**DOI:** 10.5256/f1000research.16184.r34584

**Published:** 2018-06-14

**Authors:** Henry J C de Vries

**Affiliations:** 1 University of Amsterdam, department of dermatology, Amsterdam Institute for Infection and Immunity (AI&II), Meibergdreef 9, Amsterdam, The Netherlands; 2 Public Health Service (GGD) , Amsterdam, The Netherlands

This is a nicely written paper proposing a new and refreshing hypothesis that gonorrhoea screening in a larger proportion of a certain population and subsequent treatment could induce AMR. Although the authors admit that they cannot confirm their hypothesis, they do claim to see an association in support of their claim. There is a multitude of other explanations for the association found which aren't properly discussed and more sound evidence is needed to confirm their statement. It is of interest though to report on their findings. 

 I have some comments to consider though.

1. In the method section it is stated that MSM were compared with women rather than heterosexual men to avoid the problem of misclassification of men who occasionally have sex with men but regard themselves as heterosexual. In the same fashion it is possible that heterosexual women might have sex with bisexual males and thus be exposed to the MSM pharmacoecology described here. Please consider the effect of this option in the light of choosing the control group.
2. The MIC right shift for all 3 antibiotics has decreased from 2010/11 to 2014 in the UK this is attributed to higher dosages of cephalosporins given and the addition of azi to the recommended therapy. This finding can be interpreted as an argument against the hypothesis of the authors; correct treatment of a confirmed infection does not lead to the induction of AMR since the strain is eradicated and cannot develop AMR.  
3. It is stated also that ceftriaxone's longer half-life than cefixime may have played a role in preventing MIC drift in Belgium. This is counter intuitive. A longer antimicrobial half life is associated with the induction of AMR due to the prolonged exposure of bacteria to sub therapeutic concentrations of antibiotic during re-exposure. See also: Decreased Azithromycin Susceptibility of Neisseria gonorrhoeae Isolates in Patients Recently Treated with Azithromycin. Wind CM, de Vries E, Schim van der Loeff MF, van Rooijen MS, van Dam AP, Demczuk WHB, Martin I, de Vries HJC. Clin Infect Dis. 2017 Jul 1;65(1):37-45. [Ref-1]
4. In the second paragraph of the discussion it isn't mentioned that: “This explanation stems from the insight that the intensity of exposure to antimicrobials plays a crucial role in the genesis of AMR [ref 21]. Here, the reference is misquoted, Cantas et al specifically address the non-therapeutic and low-level dosage use of antimicrobials that lead to AMR induction. This is not the case in the UK setting where MSM are treated with therapeutic dosages, and only after infection has been confirmed. 

I have read this submission. I believe that I have an appropriate level of expertise to confirm that it is of an acceptable scientific standard, however I have significant reservations, as outlined above.

## References

[1] WindCde VriesESchim van der LoeffMvan RooijenMvan DamADemczukWMartinIde VriesH: Decreased Azithromycin Susceptibility of Neisseria gonorrhoeae Isolates in Patients Recently Treated with Azithromycin. *Clinical Infectious Diseases*.2017;65(1) : 10.1093/cid/cix249 37-45 10.1093/cid/cix249 28510723

# Response

**Published:** 2018-06-19

**Authors:** Chris Kenyon

**Affiliations:** Institute of Tropical Medicine, Antwerp, Belgium

Dear Prof. de Vries

Thank you for your useful suggestions which we respond to below:

1. This is a valid concern. Repeating the analyses using heterosexual men instead of women as the control group makes little difference to the MSM vs. control MIC frequency distribution curves. This is evident if one looks at the individual GRASP reports. We can share the figures for Belgium if there is interest in this.

2. and 3.  It is true that we do not know why the right shift has declined. It is also true that a long half of an antibiotic is frequently associated with the induction of resistance. Azithromycin is a good example of this as noted in the reference you refer to. We have argued elsewhere that a N. gonorrhoeae (Ng) infection during the long declining half life of azithromycin is a plausible risk factor for inducing AMR (ref 27). This is however quite different to what is being argued here. If an antibiotic does not attain a Ng requires a free time above MIC for cephalosporins for at least 10-20 hours. If this is not attained this will place a selection pressure to develop AMR. Because the half life of cefixime is shorter than that of ceftriaxone (3.4 hours vs. 8.45 hours) there is a higher risk of not attaining the required free time above MIC and thereby selecting for AMR (doi:10.1093/jac/dkq289). Other factors such as the ratio of the Mutant Prevention Concentration to MIC ratio may also play a role but ultimately we do not know with certainty the reasons why cefixime is more selective for resistance.

What we do have good experimental evidence for however is that for a number of bug-drug combinations cefixime is more prone to AMR than other third generation cephalosporins. One of the most convincing studies of this was an in a in vitro differential selection study by Negri et al., who found that cefixime was the best selector of penicillin resistance in *Streptococcus pneumoniae* (compared to amoxicillin, cefuroxime and cefotaxime (PMID: 8141563). The mechanism underpinning this effect has not been clearly elucidated but a number of authors have speculated that it may be related at least in part to cefixime's shorter half life.  

We will add this discussion to the next version of the paper.

4. In the next version of the paper we will include the references listed below to better back up this claim. The Cantas reference should however remain as it is a useful overview of the importance of considering total antimicrobial consumption in an ecosystem perspective. The main pathway from high antimicrobial consumption to AMR is not via subtherapeutic dosing but rather factors such as antimicrobial induced changes to the resistome and microbiome which can then be taken up by Ng via mechanisms such as transformation, plasmids. This and other mechanisms are outlined in the references below as well as refs 27-29 and 32 above.

Hicks LA, Chien YW, Taylor TH, Jr., Haber M, Klugman KP, Active Bacterial Core Surveillance T. Outpatient antibiotic prescribing and nonsusceptible Streptococcus pneumoniae in the United States, 1996-2003. Clin Infect Dis. 2011;53(7):631-9. Epub 2011/09/06. doi: 10.1093/cid/cir443. PubMed PMID: 21890767.

Bronzwaer SL, Cars O, Buchholz U, Molstad S, Goettsch W, Veldhuijzen IK, et al. A European study on the relationship between antimicrobial use and antimicrobial resistance. Emerg Infect Dis. 2002;8(3):278-82. Epub 2002/04/03. doi: 10.3201/eid0803.010192. PubMed PMID: 11927025; PubMed Central PMCID: PMCPMC2732471.

Riedel S, Beekmann SE, Heilmann KP, Richter SS, Garcia-De-Lomas J, Ferech M, et al. Antimicrobial use in Europe and antimicrobial resistance in Streptococcus pneumoniae. European Journal of Clinical Microbiology & Infectious Diseases. 2007;26(7):485-90. doi: 10.1007/s10096-007-0321-5. PubMed PMID: WOS:000247930300007.

Goossens H, Ferech M, Vander Stichele R, Elseviers M, Group EP. Outpatient antibiotic use in Europe and association with resistance: a cross-national database study. Lancet. 2005;365(9459):579-87. Epub 2005/02/15. doi: 10.1016/S0140-6736(05)17907-0. PubMed PMID: 15708101.

Bruyndonckx R, Hens N, Aerts M, Goossens H, Cortinas Abrahantes J, Coenen S. Exploring the association between resistance and outpatient antibiotic use expressed as DDDs or packages. J Antimicrob Chemother. 2015;70(4):1241-4. Epub 2015/01/15. doi: 10.1093/jac/dku525. PubMed PMID: 25585511.

Klein EY, Van Boeckel TP, Martinez EM, Pant S, Gandra S, Levin SA, et al. Global increase and geographic convergence in antibiotic consumption between 2000 and 2015. Proc Natl Acad Sci U S A. 2018;115(15):E3463-E70. Epub 2018/03/28. doi: 10.1073/pnas.1717295115. PubMed PMID: 29581252; PubMed Central PMCID: PMCPMC5899442.

van de Sande-Bruinsma N, Grundmann H, Verloo D, Tiemersma E, Monen J, Goossens H, et al. Antimicrobial drug use and resistance in Europe. Emerg Infect Dis. 2008;14(11):1722-30. Epub 2008/11/04. doi: 10.3201/eid1411.070467. PubMed PMID: 18976555; PubMed Central PMCID: PMCPMC2630720.
